# The Glioblastoma CircularRNAome

**DOI:** 10.3390/ijms241914545

**Published:** 2023-09-26

**Authors:** Alexandru Tirpe, Cristian Streianu, Stefana Maria Tirpe, Anja Kocijancic, Radu Pirlog, Bianca Pirlog, Constantin Busuioc, Ovidiu-Laurean Pop, Ioana Berindan-Neagoe

**Affiliations:** 1 Research Center for Functional Genomics, Biomedicine and Translational Medicine, Iuliu Hatieganu University of Medicine and Pharmacy, 23 Marinescu Street, 400337 Cluj-Napoca, Romania; altirpe@gmail.com (A.T.); pirlog.radu@yahoo.com (R.P.); 2 The Oncology Institute “Prof. Dr. Ion Chiricuta”, 400015 Cluj-Napoca, Romania; crstreianu@gmail.com; 3 Department of Neurology, Ortenau-Klinikum Lahr, Klostenstrasse 19, 7933 Lahr, Germany; sciortea@gmail.com; 4 Department of Microbiology, Oslo University Hospital, N-0424 Oslo, Norway; akocijancic@gmail.com; 5 Department of Neurology, County Emergency Hospital, 400012 Cluj-Napoca, Romania; byanemes@gmail.com; 6 Department of Pathology, National Institute of Infectious Disease, 021105 Bucharest, Romania; busuioc.constantin@gmail.com; 7 Department of Pathology, Onco Team Diagnostic, 010719 Bucharest, Romania; 8 Department of Morphological Sciences, Faculty of Medicine and Pharmacy, University of Oradea, 410073 Oradea, Romania; drovipop@yahoo.com

**Keywords:** circRNA, glioblastoma, ncRNA

## Abstract

Glioblastoma remains one of the most aggressive cancers of the brain, warranting new methods for early diagnosis and more efficient treatment options. Circular RNAs (circRNAs) are rather new entities with increased stability compared to their linear counterparts that interact with proteins and act as microRNA sponges, among other functions. Herein, we provide a critical overview of the recently described glioblastoma-related circRNAs in the literature, focusing on their roles on glioblastoma cancer cell proliferation, survival, migration, invasion and metastasis, metabolic reprogramming, and therapeutic resistance. The main roles of circRNAs in regulating cancer processes are due to their regulatory roles in essential oncogenic pathways, including MAPK, PI3K/AKT/mTOR, and Wnt, which are influenced by various circRNAs. The present work pictures the wide implication of circRNAs in glioblastoma, thus highlighting their potential as future biomarkers and therapeutic targets/agents.

## 1. Introduction

Although there are over 100 histologically different central nervous system (CNS) tumors, glioblastoma (GBM) remains the most aggressive malignant cancer of the brain. The 2021 statistics show that the combined glioblastoma incidence for all ages is 3.23 per 100,000 people, accounting for approximately 49% of all primary malignant CNS tumors in all ages combined [1]. This poses a real problem, as current therapies lack significant efficacy, translated into a lack of considerable progression-free survival/overall survival (PFS/OS) benefit. As the incidence increases with age, statistical reports suggest that the highest incidence is between 75–84 years, and it is more common in men [1]. Current management includes surgical resection with concomitant radiochemotherapy with temozolomide (TMZ) [2].

GBM can be classified in numerous ways; first and foremost, GBMs were traditionally classified as primary (~90% of GBMs)—with no precursor lesion—and secondary, which arise from lower grade gliomas, such as anaplastic astrocytoma or diffuse astrocytoma [3]. Although histologically not distinguishable, it is essential to note that primary and secondary GBMs are essentially different, each fostering distinct alterations in their genetic and epigenetic code, which are used for both patient stratification and prognostic purposes. Furthermore, from a genetic perspective, there are a number of primary GBM-specific alterations—PTEN deletion, 7p chromosome EGFR amplification/high-rate mutation, 9p chromosome CDKNA2A-p16^INK4a^ homozygous deletion, MDM2 amplification, NF1 mutations, and even PI3KR1 homozygous deletion [3,4,5]. In contrast, secondary GBMs may harbor IDH1 or 2 (considered a mark of secondary GBMs) and TP53 mutations, along MGMT deactivation via promoter methylation [6].

In a study on 200 GBMs, four clinically relevant subtypes of GBMs were identified—classical, mesenchymal, and proneural—based on alterations in several genes—EGFR, NF1, and PDGFRA/IDH1, respectively [7]. The fourth subtype is neural, which displays neuron-related genes and is associated with oligodendrocytic and astrocytic genetic signatures. The proneural subtype is associated with a better prognosis and with oligodendrocytic genetic signatures. New insights in the glioblastoma pathogenesis are also offered by mass spectrometry proteomics approaches, which can uncover proteic signatures specific to this disease and to specific subtypes [8]. Another molecular event with clinical importance is the epigenetic deactivation of MGMT—through promoter methylation—a predictor of a higher benefit of TMZ, an alkylating agent, in GBM patients [2], as the MGMT gene encodes a protein with DNA repair capabilities that functions to remove alkyl groups from the O^6^ position of guanine [9].

Circular RNAs (circRNAs) are a type of single-stranded RNA loop molecules closed with a covalent bond [10,11]. Even though circRNAs were once considered to be mainly junk material produced by aberrant splicing [12], more recent studies show their ubiquitous-like expression in human tissues and their large implication in various human pathologies, such as multiple cancers, diabetes mellitus, chronic inflammatory diseases, and even cardiovascular diseases [11]. Concomitantly, their complex biogenesis and functions are only partially elucidated nowadays.

This review article is an integrative work that discusses the updated information on the glioblastoma circularRNAome and their supposed and proposed function, as well as the prognostic, predictive, and therapeutic potential given in glioblastoma, an intricate pathology with a poor prognosis.

## 2. Circular RNAs: Biological Considerations

CircRNAs are a rather newly discovered class of non-coding RNAs (ncRNAs) (of which some are translatable [13]) that are unique in the fact that their 3′ and 5′ ends are covalently bound, leaving no free residues [14]. These circRNAs are synthesized by backsplicing, with most circRNAs originating from protein-coding gene-derived exons that are near the 5′-end [15]. One such protein-coding gene can synthesize multiple circRNAs [14]. The main characteristics of circRNAs include their increased stability and resistance to RNAse R, and thus an increased half-life [16] in comparison to their linear counterparts, such as microRNAs.

**CircRNA biogenesis considerations.** In eukaryotic cells, mRNA usually loses introns through a spliceosome-dependent mechanism, thus producing linear RNA in a canonical splicing manner. In contrast, circRNAs are produced through a different competitive mechanism, backsplicing, which requires the addition of a 3′-5′ phosphodiester bond that covalently connects the 3′ splice site to the 5′ splice site. Backsplicing is tightly regulated by various transcription factors, RNA-binding proteins, cis acting elements, and trans acting elements [17]. Regarding the backsplicing mechanism, two models were proposed [18]—the exon-skipping model (lariat-driven circularization model) and the intron-pairing-driven model. The exon-skipping model assumes that the exon skipping produces a functional transcript and thus forms a lariat intermediate with skipped exons and introns. Secondary cleave releases the exon-intron circRNAs, exonic circRNAs, and intronic circRNAs. Several motifs can stabilize circular intronic RNAs (ciRNAs) by stimulating lariat structure formation [19]. In contrast, in the intron-pairing-driven model, long flanking introns form secondary structures in pre-mRNA via direct base pairing. These flanking introns can be modified via RNA-binding protein (RBP) binding, which helps the two splicing sites to form a loop. One such example is the MBL protein which stimulates circRNA production [16,20,21]. Additionally, circRNAs can emerge from pre-tRNA as well through the tRNA splicing endonuclease (TSEN) which recognizes the BHB motif and cleaves the anticodon loop within the pre-tRNA; these introns are then circularized via RTCB-like proteins, forming tRNA intronic circRNAs [22,23].

CircRNAs act through various mechanisms in order to achieve their functions, which are still to be fully discovered. One of the main circRNA roles is the inhibition of miRNA activity through their miRNA sponging action, with an effect on regulating gene expression [24]. Concomitantly, several studies have proven that some circRNAs can regulate transcriptional activity by direct mechanisms [25], as well as gene splicing and translation [26], considering the fact that some circRNAs may be located within the nucleus or in the cytoplasm. Concomitantly, circRNAs can interact with proteins in order to act as protein decoys and may also be implicated in peptide and epigenetic regulation [27].

## 3. Overview of CircRNAs in Glioblastoma

As expected, the ubiquitous-like aspect of circRNAs and their abundant dynamic expression within the brain [28,29], along with their relative novelty, make them the ideal candidates for investigation for new GBM biomarkers. Considering that the increased proliferation rate of GBM needs to be sustained by vascularisation, it is known that GBM is highly dependent on angiogenesis [30]. As a proliferative disease, GBM is also sustained by other main cancer-related processes, such as uncontrolled proliferation, metastasis, and metabolic alterations. From a molecular standpoint, circRNAs are largely implicated in gliomas in a number of cancer-related pathways, including MAPK [31], PI3K/AKT/mTOR [32], and Wnt [33].

Emerging data suggests that the dynamic GBM tumor microenvironment plays a crucial role in the development and progression of GBM, stimulating tumor cell survival and resistance to therapy. It consists of several cellular and non-cellular entities, such as extracellular matrix, exosomes, and various molecules. Concomitantly, the cellular niche contains specific cells from the neural repertoire—neurons, oligodendrocytes, astrocytes—as well as microglia, tumor-associated macrophages (TAMs), and tumor-infiltrating lymphocytes (TILs) [34,35]. The crosstalk of circular RNAs with the GBM tumor microenvironment is extensive and currently not characterized in its entirety. In terms of interactions, circular RNAs have been proven to interact with cancer-associated endothelial cells, for example, circ-DICER1 [36], promoting angiogenesis. Hypoxia, as an integrative part of TME, plays a role in cancer development and progression [37]. CircDENND2A expression was induced by hypoxia, promoting glioma cell migration and invasion [38]. Furthermore, when it comes to the crosstalk between circRNAs and the ECM, circCLSPN modulates MMP2 and MMP9 expression in GBM [39]. Oxidative stress may play a role in GBM as well [40,41]. All these points serve as arguments that prove the biological complexity of GBM. This, in turn, translates to a more difficult clinical therapeutic management, with a poor survival.

This section serves as an integrative work that presents the current scientific knowledge on the implication of circRNAs in glioblastoma genesis and progression.

### 3.1. Mechanism of Action of circRNAs in Glioblastoma

As previously mentioned, circRNAs act through various mechanisms to influence glioblastoma. These functions pertain to the specific structure of each circRNA.

First and foremost, one of the main functions of circRNA is the miRNA sponging action, which is dependent on the miRNA response element (MRE) region found within the circRNAs. These can also influence the miRNA–mRNA interaction [42], thus affecting downstream protein production. As such, circRNAs competitively bind specific miRNAs through their MREs and thus directly act through a sponge adsorption mechanism in order to achieve their function [43]. The following section will present numerous circRNAs that act as miRNA sponging agents in order to influence the fate of GBM cancer cells. Concomitantly, circRNAs can interact with miRNAs and transport the circRNA–miRNA complex to other sites, thus allowing miRNAs to exert their roles in other spatial locations [43].

Another important function of circRNAs consists of their ability to interact with specific proteins via RBPs and exert their effects [44]. Of note is that RBPs are a type of proteins that mediate various RNA processes, including maturation, localization, transport, and translation [45], and are dependent on the type of cell, circRNA, and the microenvironment biology [44]. Furthermore, the interaction between circRNA and proteins may influence protein function and expression and, in contrast, circRNA biogenesis and fate, thus marking their bidirectional role. It is worth mentioning that there are reports regarding the regulatory role of circRNAs in the ribosome–protein pair [46]. In addition, many circRNAs can interact with proteins and act as a sponge, thus regulating their effects; for example, circ-Amotl1 may interact with AKT1 and PDK1 and alter their function [47]. Concomitantly, circRNAs can function as protein decoys as well, altering proteins’ function. Using the same circ-Amotl1 as an example, this circRNA interacts with c-Myc and sequesters c-Myc within the nucleus, thus upregulating its targets and increasing cell proliferation and oncogenic features [48]. Other circRNA functions include protein recruitment and circRNA translation, as many circRNAs are circularized from protein-coding exons [44].

**Figure 1:**
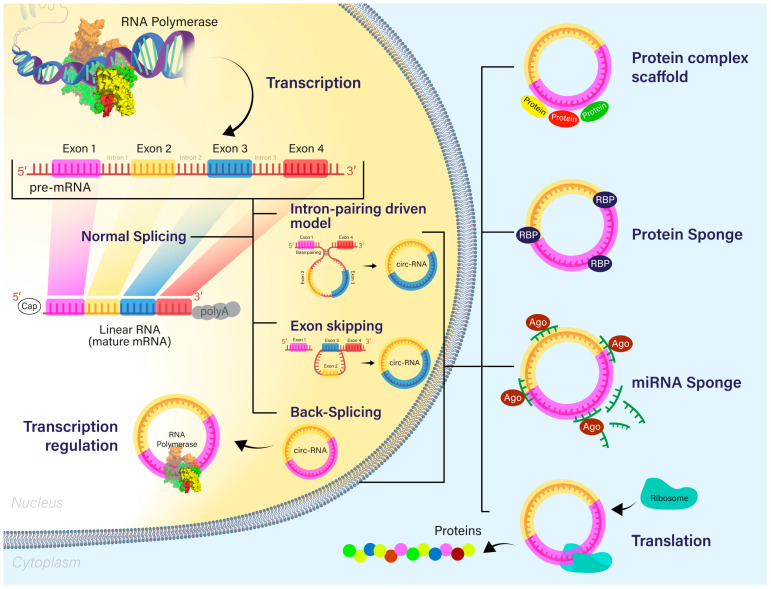
The biogenesis of circRNA through various mechanisms—direct backsplicing, exon skipping, and the intron-pairing-driven model—from an intermediary transcript. The main functions are represented—miRNA sponging, protein sponging through RBPs, and the formation of the protein scaffold complex. Some circRNAs that encode proteins may be further translated into proteins.

Nuclear circRNAs can modulate gene expression transcriptionally and post-transcriptionally [15]. As a short example, ci-ANKRD52 interacted with RNA Pol II and acted as a positive regulator of Pol II transcription [19]. Furthermore, when considering the post-transcriptional activity, more exactly selective splicing, Ashwal-Fluss et al. showed that circMBL may be able to compete with the MBL pre-mRNA and downregulate the functional MBL mRNAs [20]. Figure 1 presents the biogenesis and main functions of circRNAs.

### 3.2. Circular RNAs Are Implicated in GBM Cancer Cell Proliferation, Survival, Migration, Invasion and Metastasis

As expected, the implication of circRNAs in GBM repertoire is vast. CircRNAs are implicated in glioblastoma cancer cell proliferation and survival, migration, invasion, and metastasis. This chapter systematically discusses the large implications of recently discovered circular RNAs in glioblastoma, taking into consideration glioblastoma cancer cell proliferation, survival, migration, invasion, and metastasis, as the majority of the studies cited within this subchapter have grouped these cancer processes altogether. Table 1 presents a selection of the most recent circular RNAs identified in glioblastoma research on these processes.

### 3.3. Circular RNAs as Regulators of GBM Neoangiogenesis

While the previous subchapter considered cancer cell proliferation, survival, and metastatic features, it is without doubt that circRNAs are involved in the development of new vasculature that sustains the growth of the tumor at the cellular level. Neoangiogenesis assumes that new vasculature develops from an existing network of vasculature [93,94]. The present subchapter systematically takes into consideration recent circular RNAs that modulate the neoangiogenic process. As such, Table 2 presents the most recent circular RNAs that interact with the neoangiogenic machinery.

### 3.4. Circular RNAs Are Implicated in Metabolic Reprogramming and Therapeutic Resistance

Naturally, the wide repertoire of circRNAs includes the alteration of metabolism and therapeutic resistance. A recent study suggested that circHEATR5B expression level was low in GBM tissues and cells [100]. The authors showed that overexpression of both circHEATR5B and ZCRB1 led to aerobic glycolysis and cell proliferation suppression. Furthermore, HEATR5B-881aa, a protein encoded by circHEATR5B, reduced JMJD5 stability. JMJD5 knockdown inhibited cancer cell proliferation and glycolysis in GBM cancer cells via increasing PKM2 activity. Thus, Song et al. showed that the ZCRB1/circHEATR5B/HEATR5B-881aa/JMJD5/PKM2 axis is involved in the metabolic reprogramming of glioblastoma and may act as a potential target [100].

In regards to therapeutic resistance, a study by Li et al. [101] showed that circ_0043949, a circRNA derived from the BRCA1 gene, was differentially expressed in TMZ-resistant GBM, whilst being aberrantly upregulated in other studies on secondary TMZ-resistant GBM [102]. Mechanistically, Li et al. found that miR-876-5p levels are low in TMZ-resistant GBM samples and cell lines, and that circ_0043949 functions as a miR-876-3p sponge. MiR-876-3p knockdown impairs circ_0043949 impact on TMZ resistance, suggesting that the circ_0043949 effect upon TMZ resistance is miR-876-3p-dependent. Furthermore, the authors concluded that circ_0043949 may mediate TMZ resistance in GBM via miR-876-3p/ITGA1 axis [101].

Furthermore, in a combined in vitro and in vivo study [103], the authors found that circ_0060055 may interact with miR-197-3p and positively regulate apoptosis inhibitor 5 (API5). In the Yuan study, a miR-197-3p inhibitor abolished circ_0060055 knockdown effects on cell growth, invasion, and radiosensitivity. In contrast, the presence of miR-197-3p suppressed cell progression whilst improving the radiosensitivity effect. API5 overexpression was found to cancel the miR-197-3p-derived effects, proving the implication of circ_0060055/miR-197-3p/API5 axis in GBM progression and radiosensitivity [103].

Another study [104] found that circAKT3 expression in GBM tissues is lower than adjacent normal tissue, and this circRNA encodes a novel AKT3-174aa protein. This AKT3-174aa protein, when overexpressed, proved to decrease the proliferation, radiation resistance, and in vivo tumorigenicity of GBM cells. Furthermore, the authors suggested that AKT3-174aa may ultimately negatively regulate the PI3K/AKT signal intensity via the pPDK1 competitive interaction [104].

A study by Yuan et al. [105] found that a low hsa_circ_0072309 level in glioma patients predicts poor prognosis. Hsa_circ_0072309 stimulated GBM sensitivity to TMZ and promoted autophagy via the p53 pathway, inhibiting p53 wild-type ubiquitination and increasing its stability. The authors suggest that miR-100 mediated hsa_circ_00742309-dependent p53 regulation [105]. Additionally, hsa_circ_0072309 levels were downregulated in GBM as well, and hsa_circ_0072309 inhibited GBM proliferation and invasion, with a potential association with HSP27 [106].

Regarding cisplatin resistance, in a study by Luo et al. [107], circ_PTN sequestered miR-542-3p which, in turn, upregulated PIK3R3 and activated PI3K/AKT signaling. These effects had a role in enhancing GBM cells’ resistance to cisplatin. In vivo, the authors found that circ_PTN silencing inhibited the GBM tumors’ cisplatin resistance [107].

Additionally, a study by Wei et al. found the circASAP1 expression to be significantly upregulated in recurrent GBM and GBM cell lines resistant to TMZ, enhancing cell proliferation and TMZ resistance. Mechanistically, circASAP1 sponged miR-502-5p and increased NRAS expression; by downregulating circASAP1, TMZ sensitivity was restored [108].

All these arguments prove the wide implication of circRNAs in the GBM fate, from development to progression, and from cancer cell proliferation, survival, invasion, metastasis to neoangiogenesis, metabolic alteration induction, and therapeutic resistance.

## 4. Perspectives on circRNAs in Translational Medicine

This paper systematically describes the current view of circRNAs in GBM. Being rather recent entities, the real-world clinical use of circular RNAs is yet to be determined. As previously discussed, circRNAs act through various mechanisms to influence glioblastoma fate. We would like to highlight the miRNA sponging effect and the circRNA–protein interaction as essential in GBM genesis and progression.

Recent studies show that circRNAs may play a prognostic role in various cancers—arguably, in some, even a diagnostic role—including glioblastoma [55,56,109,110,111]. Naturally, circRNAs may be exploited as treatment options. Many methods used to target circRNAs were reviewed by He et al., of which we mention the CRISPR/Cas9-mediated and CRISPR/Cas13-mediated circRNA knockdown via different mechanisms, the circRNA knockdown via cre-dependent short hairpin RNA, siRNA/shRNA that target the backsplice junction leading to cleavage, and circRNA plasmids that, on the contrary, lead to circRNA overexpression [112]. It is worth mentioning that siRNA-mediated circRNA knockdown is now the most feasible method of circRNA inhibition [112]. At the time of submitting the current article, there are no clinical trials regarding the potential of circular RNAs in GBM. Future studies should focus on revealing the various circRNA functions in GBM and thoroughly characterizing these circRNAs, as well as developing new methods and perfecting current ones regarding the difficult access to this specific type of tumor.

## 5. Concluding Remarks

The present article is an integrative work on the role of circRNAs in GBM. As a disease with poor prognosis and limited therapeutic options, there is an urgent need to uncover new cellular vulnerabilities that can be exploited in novel therapeutic approaches. Herein, we have critically appraised the recent advancements in understanding GBM and circRNA biology and thoroughly characterized their involvement in GBM—from their miRNA sponging effect to their properties as agents interacting with proteins.

As circRNAs are implicated in various GBM cancer processes, ranging from GBM cell proliferation to cell survival, migration, invasion, metastasis, neoangiogenesis, metabolic reprogramming, and even therapeutic resistance, it is abundantly clear that these entities are worth exploiting. Naturally, there are a number of advantages in studying circRNAs as potential biomarkers and therapeutic targets/agents. In the latter case, circRNAs are much more stable than other types of linear RNA due to their covalent circularization and thus are less enzymatically degradable. Another advantage is their potential to be used in vaccine development and nucleic acid drug development, where heat stability is essential. In comparison with their mRNA counterparts, a circRNA-derived vaccine proved to have increased heat stability [113]. In contrast, the in-detail study of circRNAs presents a number of challenges and limitations. The clinical use of circRNAs necessitates a vector—for example, an adeno-associated viral vector (rAAV) was used in a study, which may cause immune adverse events [114]. The synthetic process of circRNAs may be more laborious and with a somewhat lower yield in comparison to the linear counterparts, as the circularization process may generate considerable byproducts [113]. At the time of submitting the present article, there are no clinical trials involving circRNAs as therapeutic agents in GBM.

The current understanding of circRNAs marks them as entities worthy of further exploration, and future studies should focus on thoroughly characterizing the function and the potential of circular RNAs in clinic.

## Figures and Tables

**Table 1:** Recently identified circular RNAs in glioblastoma cancer cell proliferation, survival, migration, invasion, and metastasis.

circRNA	Target	Proposed Function	Mechanism of Action	Ref.
circSKA3	miR-1	oncogenic	CircSKA3 increased miR-1 gene methylation and thus silenced miR-1, reducing its inhibitory roles upon cell proliferation.	[49]
CircXPO1	miR-7-5p	oncogenic	CircXPO1 is significantly upregulated in GBM. In vitro circXPO1 knockdown inhibited cell proliferation and migration. In contrast, overexpression of circXPO1 increased the malignant traits of GBM. CircXPO1 inhibits the tumor suppressive miR-7-5p, which negatively regulated RAF1, thus promoting malignant traits.	[50]
CM21D	miR-21	tumor suppressive	CM21D is a synthetic miRNA decoy created by tRNA splicing in vitro with inhibitory effects upon cell proliferation, migration, and cell cycle and an inductor of apoptosis.	[51]
circPTK2	miR-23a	tumor suppressive	CircPTK2 may inhibit GBM cancer cell invasion and migration via miR-23a maturation inhibition.	[52]
circENTPD7	miR-101-3p	oncogenic	CircENTPD7 sponged miR-101-3p and regulated ROS1 expression, promoting proliferation and glioblastoma cancer cell motility.	[53]
circPARP4	miR-125a-5p	oncogenic	CircPARP4 may promote GBM cell proliferation, migration, invasion, and EMT through the circPARP4/miR-125a-5p/FUT4 axis.	[54]
circLGMN	miR-127-3p	oncogenic	In the Chen study, circLGMN sponged miR-127-3p and hampered miR-127-3p-mediated LGMN mRNA degradation, thus increasing LGMN expression; circLGMN overexpression stimulated GBM malignancy in vivo.	[55]
circASPM	miR-130b-3p	oncogenic	CircASPM levels are increased in GBM. In the Hou study, circASPM stimulated both tumorigenesis and GSCs proliferation in vitro and in vivo via miR-130b-3p sponging. Via miR-130b-3p sponging, circASPM upregulated E2F1 expression and acted on GSCs’ proliferation.	[56]
CircNDC80	miR-139-5p	oncogenic	CircNDC80 expression was increased in GBM tissues. CircNDC80 sponges miR-139-5p and affects ECE1 expression, thus presenting as a pro-oncogenic entity. This circRNA sustains stemness and promotes cell proliferation, migration, and invasion.	[57]
circFLNA	miR-199-3p	oncogenic	CircFLNA was upregulated in GBM tissues and cells, which were associated with a poorer prognosis. CircFLNA/miR-199-3p axis may play a role in the proliferative and invasive features of GBM.	[58]
circ_0001588	miR-211-5p	oncogenic	Circ_0001588 is upregulated in GBM tissues and human glioma cells and correlated with poor survival. Circ_0001588 sponged miR-211-5p and positively regulated YY1, stimulating GBM proliferation, migration, and invasion.	[59]
CircFGFR1	miR-224-5p	oncogenic	CircFGFR1 sponges miR-244-5p and thus increases CXCR4 expression, promoting glioma growth.	[60]
CircHECTD1	miR-320-5p	oncogenic	CircHECTD1 functioned as a ceRNA and interacted with miR-320-5p with SLC2A1 as a target. CircHECTD1 expression promoted proliferation and migration in vitro and tumor growth in vivo.	[61]
circSERPINE2	miR-324-5p miR-361-3p	tumor suppressive	CircSERPINE2 may sponge miR-324-5p and miR-361-3p, thus promoting BCL2 expression. As such, circSERPINE2 may act as an inhibitor of GBM cell proliferation.	[62]
CircRNA-SMO	miR-326	oncogenic	In the Wu study, circRNA-SMO was found to be upregulated in GBM tissues and cells. CircRNA-SMO has a sponging effect upon miR-326, thus upregulating CEP85 expression, leading to increased proliferation and migration.	[63]
circCD44	miR-326 miR-330-5p	tumor suppressive	LRRC4 promoted circCD44 generation by inhibiting SAM68-CD44 pre-mRNA interaction. CircCD44 was found to be downregulated in GBM tissues. CircCD44 may sponge miR-326 and miR-330-5p and thus regulate SMAD6, with an effect on tumor growth.	[64]
Circ_0012381	miR-340-5p	oncogenic	Circ_0012381 expression was found to be increased in irradiated GBM cancer cells, whilst exosomes derived from these cells significantly induced M2 polarization of microglia. Mechanistically, circ_0012381 functions as a miR-340-5p sponge, thus increasing ARG1 expression; these M2-polarized microglia promote GBM cancer cell growth via CCL2/CCR2 axis.	[65]
circARID1A	miR-370-3p	oncogenic	CircARID1A stimulates GBM cancer cell migration and invasion via the miR-370-3p/TGFBR2 axis.	[66]
Circ_0000741	miR-379-5p	oncogenic	In SAHA-tolerant GBM cells, circ_0000741 silencing reduced HDAC inhibitor tolerance, inhibited invasion and proliferation, and induced apoptosis. Concomitantly, circ_0000741 absence enhanced drug sensitivity in vivo in GBM. Circ_0000741 may sponge miR-379-5p and thus affect TRIM14.	[67]
CircGLIS3	miR-449c-5p	oncogenic	CircGLIS3 positively regulates GLIS3 and CAPG via miR-449c-5p sponging to promote proliferation and inhibit apoptosis.	[68]
circCDC45	miR-485-5p	oncogenic	CircCDC45 targeted miR-485-5p and thus positively regulated CSF-1 expression, affecting GBM cell proliferation, migration, and invasion.	[69]
circZNF652	miR-486-5p	oncogenic	circZNF652 acts as a miR-486-5p sponge and thus upregulates SERPINE1 expression in GBM cells. In the Liu study, circZNF652 knockdown reversed the malignant phenotypes in GBM cells; the authors suggested that the circZNF652/miR-486-5p/SERPINE1 axis may play a role in tumorigenesis, cell growth, migration, invasion, and EMT.	[70]
CircBFAR	miR-548b	oncogenic	The circBFAR/miR-548b/FoxM1 axis regulates GBM proliferation and invasion.	[71]
CircRFX3	miR-587	oncogenic	CircRFX3 functions as a ceRNA by sponging miR-587 and alters PDIA3, which, in turn, regulates the Wnt/β-catenin pathway.	[72]
circMELK	miR-593	oncogenic	CircMELK was upregulated in GBM and sponged miR-593, thus controlling GSC maintenance and GBM mesenchymal transition.	[73]
hsa_circ_0006168	miR-628-5p	oncogenic	Hsa_circ_0006168 was upregulated in GBM tissues and cells. Hsa_circ_0006168 sponged miR-628-5p; Hsa_circ_0006168 knockdown delayed xenograft tumor growth in vivo and lowered Ras and pERK1/2 expression both in vitro and in vivo.	[74]
hsa_circRNA_0043278	miR-638	oncogenic	Hsa_circRNA_0043278 knockdown inhibited GBM cancer cell in vitro migration, proliferation and invasion, as well as in vivo tumorigenesis. hsa_circRNA_0043278 sponged miR-638 in GBM and upregulated HOXA9, thus activating Wnt/β-catenin signaling.	[75]
circABCC3	miR-770-5p	oncogenic	Interestingly, in the Zhang study, circABCC3 expression was lower in stage I + II GBM and higher in stage III GBM tissues. CircABCC3 sponged miR-770-5p, while its absence inhibited the PI3K/AKT pathway, along with cell proliferation, migration, invasion, and tube formation and induced cell apoptosis.	[32]
CircPIK3C2A	miR-877-5p	oncogenic	CircPIK3C2A expression promoted GBM cell proliferation and invasion. Mechanistically, circPIK3C2A sponged miR-877-5p, functioning as a competitive endogenous RNA (ceRNA) and modulating FOXM1 expression.	[76]
CircPTPRF	miR-1208	oncogenic	In the combined in vitro and in vivo Zhou study, circPTPRF functions as a miR-1208 sponge and thus upregulates YY1 expression. This promotes GBM cancer cell proliferation, invasion, and neurosphere formation.	[77]
Circ-AHCY	miR-1294	oncogenic	Circ-AHCY silencing inhibited GBM cell proliferation in both in vitro and in vivo experiments. Mechanistically, circ-AHCY activates the Wnt/β-catenin pathway by miR-1294 sequestration and MYC upregulation. EIF4A3 recruitment by circ-AHCY stabilizes TCF4 mRNA leading to increased TCF4/β-catenin stability, which increases circ-AHCY transcriptional activity.	[78]
CircPOLR2A	miR-2113	oncogenic	CircPOLR2A is upregulated in GBM cells. Mechanistically, circPOLR2A functioned as a miR-2113 sponge, thus positively regulating POU3F2 expression. In turn, POU3F2 activated SOX9 transcription and modulated GBM cancer cell proliferation and apoptosis.	[79]
circ-METRN	miR-4709-3p	oncogenic	Low-dose-radiation-induced exosome-derived circ-METRN acted via miR-4709-3p/GRB14/PDGFRα pathway to promote glioblastoma progression and radioresistance.	[80]
Circ-0010117	miR-6779-5p	oncogenic	In the combined in vitro and in vivo Yang study, circ-0010117 was downregulated in GBM tissues. Circ-0010117 acts via miR-6779-5p/SPEN to modulate-promote GBM cancer cell aggressiveness; circ-0010117 overexpression suppresses tumorigenesis in nude mice.	[81]
CircADAMTS6 (hsa-circ-0072688)	ANXA2	oncogenic	CircADAMTS6 is upregulated in hypoxic microenvironments; the hypoxic TME upregulates circADAMTS6 expression through AP-1 and TDP43. Next, circADAMTS6 recruits and stabilizes ANXA2, thus accelerating GBM progression.	[82]
circ_0000512 (circRPPH1_025, circRPPH1)	Not mentioned	oncogenic	In the in vitro Xue experiment on U87 cells, circRPPH1_025 promoted GBM cancer cell proliferation, migration, and invasion—EMT.	[83]
	ATF3	oncogenic	In the combined in vitro and in vivo Xu study, circRPPH1 was upregulated in GSCs. UPF1 stabilizes circRPPH1, which modulates ATF3 to further transcribe UPF1 and Nestin in a loop. This axis maintains GSC self-renewal via TGF-β activation.	[84]
circ-E-Cad	EGFR through C-E-Cad	oncogenic	C-E-Cad is a protein encoded by circ-E-Cad and activated EGFR by CR2 domain association, independent of EGF. The authors found that C-E-Cad inhibition enhanced anti-EGFR therapeutic strategies in GBM.	[85]
CircMMD	FUBP1	oncogenic	CircMMD had high expression levels in GBM and indicated a poor prognosis. In the Xu study, circMMD levels were reduced by tumor treating fields (TTF), with a concomitant increase in TTF-induced apoptosis. Low circMMD levels stimulated FUBP1–FIR interaction with decreased DVL1 transcription. Low circMMD levels may promote miR-15b-5p activity and degrade FZD6. Low DVL1 and FZD6 expression suppressed Wnt/β-catenin activation.	[86]
circHGF	HGF/c-MET	oncogenic	CircHGF RNA encodes C-HGF, and this protein variant is highly expressed in GBM compared to normal brain tissue and is secreted by GBM cells; C-HGF activates c-MET receptor in vitro in PDX GBM cell lines; C-HGF knockdown leads to inhibitory effects upon cell growth, motility, and cancer cell invasiveness.	[87]
CircSQSTM1 (hsa_circ_0075323)	p62-mediated autophagy	oncogenic	CircSQSTM1 depletion in GBM cells impairs autophagy, leading to increased p62 and decreased LC3B levels. CircSQSTM1 inhibition in vitro led to a significant inhibition of cancer cell proliferation and invasion.	[88]
CircLRFN5	PRRX2	tumor suppressive	CircLRFN5 is downregulated in GBM. CircLRFN5 binds PRRX2; PRRX2 upregulates GCH1 which suppresses ferroptosis via BH4. CircLRFN5 overexpression has inhibitory effects upon tumorigenesis, cell viability, stemness, proliferation, and neurosphere formation through a ferroptosis-dependent mechanism.	[89]
circ-SMO	SHH signaling via SMO-193aa	oncogenic	SMO-193aa attenuates SHH signaling intensity in brain cancer stem cells, as well as proliferation in vitro and tumorigenicity in vivo.	[90]
CircKPNB1	SPI1	oncogenic	CircKPNB1 was found to be overexpressed in GBM and functions to regulate SPI1 stability and SPI1 nuclear translocation; SPI1 acts via TNFα/NFκB to stimulate malignant phenotype. Thus, circKPNB1 overexpression stimulates GBM cancer cell viability, proliferation, stemness, invasion, and neurosphere formation.	[91]
circSMARCA5	N/A	N/A	CircSMARCA5 and circHIPK3 were less abundant in serum extracellular vesicles (sEV) from GBM patients compared to controls. GBM may be differentiated from controls via circSMARCA5 and circHIPK3 sEV (accuracy data within cited article). Combining preoperative NLR, PLR and LMR ratios with expression of sEV-derived circSMARCA5 and circHIPK3 improved GBM diagnostic accuracy of these markers with AUC 0.901 [95% CI, 0.7912–1.000].	[92]
circHIPK3		N/A		

**Table 2:** The modulation of neoangiogenic machinery by circular RNAs.

circRNA	Target	Proposed Function	Mechanism of Action	Ref.
circSMARCA5	miR-126-3p miR-515-5p	tumor suppressive	CircSMARCA5 targets miR-126-3p, which regulates cancer cell migration and invasion, as well as angiogenesis. CircSMARCA5 may also regulate angiogenesis via regulating VEGFA pre-mRNA alternative splicing through SRSF1 tethering.	[95]
circPOSTN	miR-219a-2-3p	oncogenic	CircPOSTN is overexpressed in GBM. In the combined in vitro and in vivo Long study, the authors identified a new circPOSTN/miR-219a-2-3p/STC1 axis that stimulated VEGFA secretion and thus neovascularization. CircPOSTN may also play a role in GBM cancer cell proliferation and migration.	[96]
circPITX1	miR-584-5p	oncogenic	CircPITX1 knockdown in functional experiment suppressed GBM angiogenesis, proliferation, migration, and tumor growth in vivo. The circPITX1/miR-584-5p/KPNB1 axis may regulate GBM progression processes.	[97]
circVPS18	miR-1229-3p	oncogenic	In the combined in vitro and in vivo Huang study, circVPS18 knockdown inhibited GBM progression, including cancer cell proliferation, migration, invasion, and even angiogenesis. CircVPS18 promoted GBM progression via miR-1229-3p/BCAT1 axis.	[98]
circKIF18A	FOXC2	oncogenic	In the Jiang experiment, transportation of exosomal circKIF18A into human brain microvessel endothelial cells (hBMECs) promoted GBM angiogenesis through a M2-GAM-dependent mechanism. CircKIF18A can stabilize and promote nuclear translocation of FOXC2 in hBMECs and modulate ITGB3, CXCR4, and DLL4 via FOXC2. Concomitantly, FOXC2 can activate PI3K/AKT and thus stimulate GBM angiogenesis.	[99]
